# Mapless Navigation Based on 2D LIDAR in Complex Unknown Environments

**DOI:** 10.3390/s20205802

**Published:** 2020-10-14

**Authors:** Kai Yan, Baoli Ma

**Affiliations:** The Seventh Research Division, Beihang University, Beijing 100191, China; yankai2016@buaa.edu.cn

**Keywords:** local path planners, complex environments, 2D LIDAR, Turtlebot2

## Abstract

This paper presents a novel approach for navigation in complex and unknown environments. At present, existing local path planners whose control output is the mapping of current sensor data have been widely studied. However, these methods cannot really solve the problem of being trapped by obstacles. We analyzed the reasons and made improvements, and finally our approach can avoid being trapped in complex environments. The proposed method is based on 2D LIDAR. A central part of the approach is finding out gaps in the environment by analyzing sensor data. Then, we choose one of the gaps we find as the sub-goal. Linear and angular velocities are provided by the approach considering nonholonomic mobile robots. The method does not rely on global planners and environment maps. Therefore, it has the advantages of low computational complexity and fast response, which is of great significance to robots with low computing power; it will also help to reduce the manufacturing cost of robots. In addition, simulations and real tests were performed using the Turtlebot2 robotic platform. Successful results are achieved in both simulations and experimental tests.

## 1. Introduction

The ability of autonomous navigation is important for the application of mobile robot systems. Robots should be able to reach the goal position safely among unknown environments. Excellent navigation performance is the basic premise on particular tasks. After decades of research, significant results have been achieved. Until now, robot navigation approaches can roughly be divided into two categories: global and local. The two types of methods are completely different from the research perspective, and they have their advantages and disadvantages.

### 1.1. Global Approaches

The first type is also called global path planning or deliberative navigation. The geometric path from the current position of the robot to the target is obtained using an environment map. There are lots of approaches to address path planning in the literature, such as artificial potential field (APF) [1], A-star($A^{*}$), D-star($D^{*}$) [2], rapidly exploring random trees (RRT) [3], and probabilistic roadmaps (PRMs) [4].

The advantages of global approaches are that the computed path can be optimal in terms of Euclidean distance traveled, and the robot will not be trapped in any environments (if paths exit). However, for most applications, the global world model is changeable or not available. Computing a path off-line is problematic. Under such circumstances, to reach the goal, updating the map and replanning the path repeatedly is required combined with the simultaneous location and mapping (SLAM) technology, which requires large amounts of computational resources. With the increase of map size, computational complexity increases exponentially. Therefore, global approaches are computationally expensive and hardly suit real-time implementation for robots with lower computing power.

### 1.2. Local Approaches

The local approach is also known as obstacle avoidance or reactive navigation. In every control cycle, the current control command of the robot is the mapping of the on-line sensory data. The key advantages of local techniques over global ones lie in their low computational complexity and fast response. Local approaches can effectively avoid collisions with obstacles. However, pure local approaches are still challenged by complex and cluttered environments in which the robot may be trapped and cannot achieve its goal. The primary requirement of a navigation approach is to reach the target without collision, while other existing local approaches are not guaranteed to reach the goal every time. Therefore, existing local approaches are difficult to navigate independently in a complex environment. Normally, local approaches are combined with global methods to form a hybrid structure [5,6,7,8].

The paper is organized as follows: Section 2 describes related work and the our research. Section 3 describes in detail the proposed navigation approach. In Section 4, simulations and real tests are discussed using the Turtlebot2 robotic platform, and successful results are achieved. Section 5 and Section 6 provides discussions and conclusions.

## 2. Related Work

Decades of efforts on local approaches have lead to some satisfactory algorithms. In the work of [1], Khatib puts forward the concept of artificial potential field (APF). The main idea is that the movement of the robot is the result of forces of two virtual potential fields. The surrounding environment exerts a repulsive force on the robot and the target exerts an attractive force on the robot. The robot heading is the same as the direction of the resultant force. The linear velocity of the robot is related to the magnitude of the resultant force.

In [9], the vector field histogram (VFH) is proposed, which uses a two-dimensional occupancy grid as an environment model. The model is updated continuously using the range data sampled by on-board ultrasonic proximity sensors. After a data-reduction process, a polar histogram of the environment around the robot is obtained using the occupancy information. Within the histogram, all openings that allow the robot to pass through are found. Then a cost function is applied to choose the optimal opening with the lowest cost. The travel direction is then aligned with the direction of the chosen opening. In the improved version VFH+ [10], the robot’s trajectories based on its kinematic model is taken into account and thus the enhanced method can reduce the risk of collision.

The dynamic window approach (DWA) [11] can generate a smooth trajectory by selecting the motion commands in velocity space, which consists of translational and rotational velocity. Each velocity uniquely corresponds to a trajectory. Obstacles on the occupancy grid map can impose restrictions on the velocities. The objective function is applied to trade-off velocity, clearance, and target heading, then the velocity that maximizes the objective function is selected.

However, none of the three local methods mentioned above can cope with the complex environment. They may be trapped by obstacles and cannot reach the target. As far as I know, only a family of bug algorithms [12] is proved to be convergent [13]. The bug algorithms navigate the robot by following the obstacle boundary if an obstacle is encountered. However, the path generated by this simple behavior can be much longer than the optimal path.

Is it possible to navigate in complex environments using simple local approaches? In [14], the intelligent navigation behaviors of animals are believed to be caused by simple local approaches. Think carefully about how we walk ourselves: While walking, we first find out the direction of movement or set a temporary target in front of us. We do not plan a complete geometric path in our minds during the journey to the target. Our behavior is just a response to the surrounding environment. It seems like that the way humans navigate is a local approach in most cases. We were never trapped in simple obstacles, such as U-shaped obstacles, but this is a big problem for robots using the local algorithm.

Inspired by human navigation behaviors, this paper extends earlier work [15] and overcomes the shortcomings of existing local approaches, then proposes a new local navigation strategy. Our approach differs from previous approaches in that: (a) it achieves the goal without a global planner, but will not be trapped in complex environments. It means that the proposed approach can accomplish the navigation task independently and has the advantages of low computational complexity and fast response; (b) there is no need to maintain an environment map, which are required for all global approaches and most local approaches; (c) the generated path is close to the optimal one. Besides, the algorithm considers the size and kinematics of the robot and has no restriction on the shape of obstacles. Therefore, our approach can make the robot with low computing power complete complex navigation tasks.

## 3. Method Description

**Figure 1:**
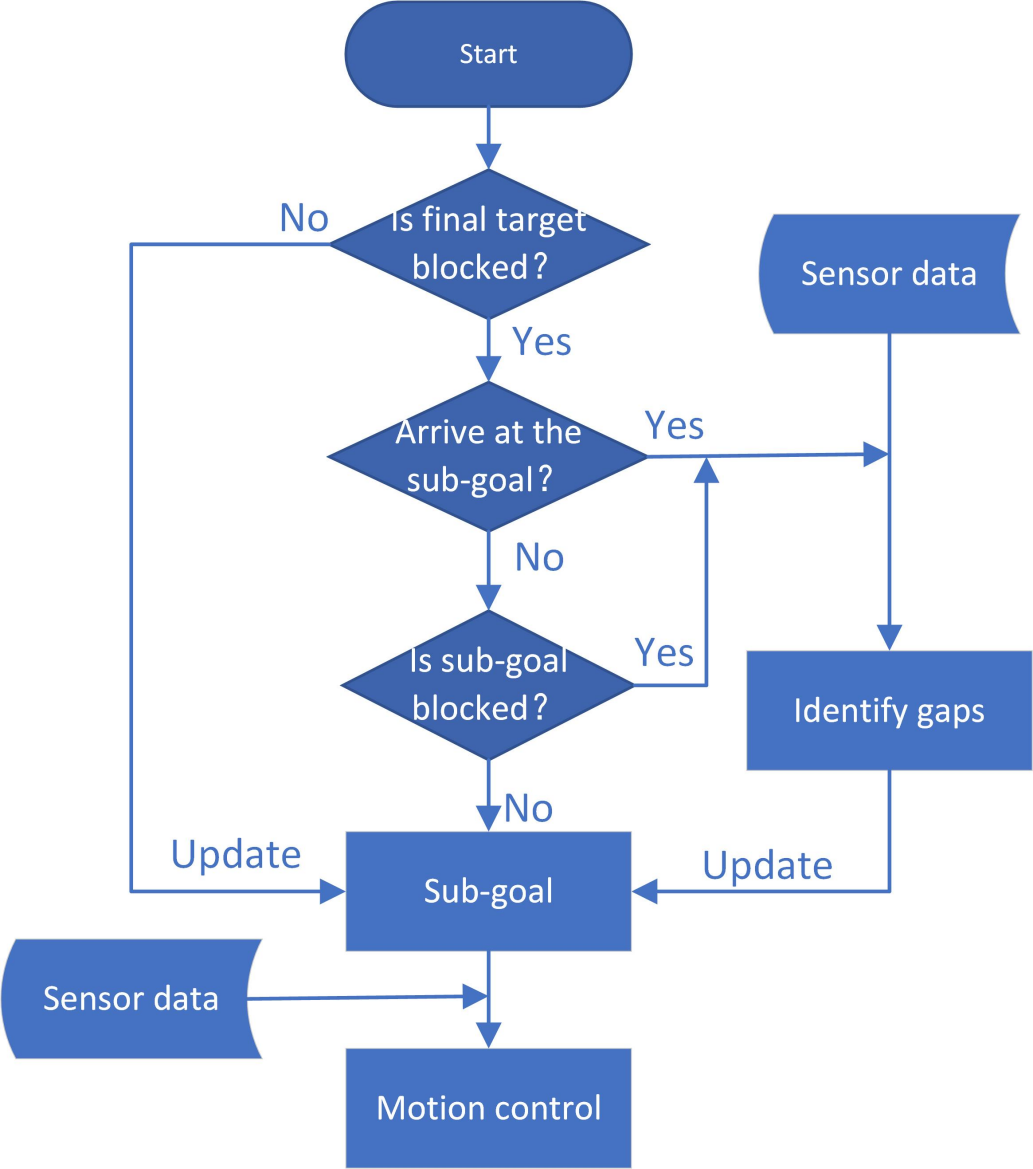
Flow diagram of the approach in one time interval.

A major part of the proposed approach is identifying gaps of the environment based on 2D LIDAR [16]. The method of identifying the gap is described in detail below. Finding a gap means that there is a large enough free space near the gap to allow the robot to pass. Then, choose one of the gaps we found as the new sub-goal, considering positions of the final target and previous sub-goals. According to the sub-goal and real-time sensor information, the robot’s motion control is obtained. The specific method is described below. Compared with existing local algorithms that directly map the sensor data to the on-line control command, the proposed algorithm takes into account not only current sensor data but also other relevant information—the previous sub-goals. This is the key factor that keeps our algorithms from getting trapped. In every time interval, the navigation architecture is illustrated in Figure 1. The process iterates until the robot reaches the final goal.

### 3.1. Find Gaps of the Environment

**Figure 2:**
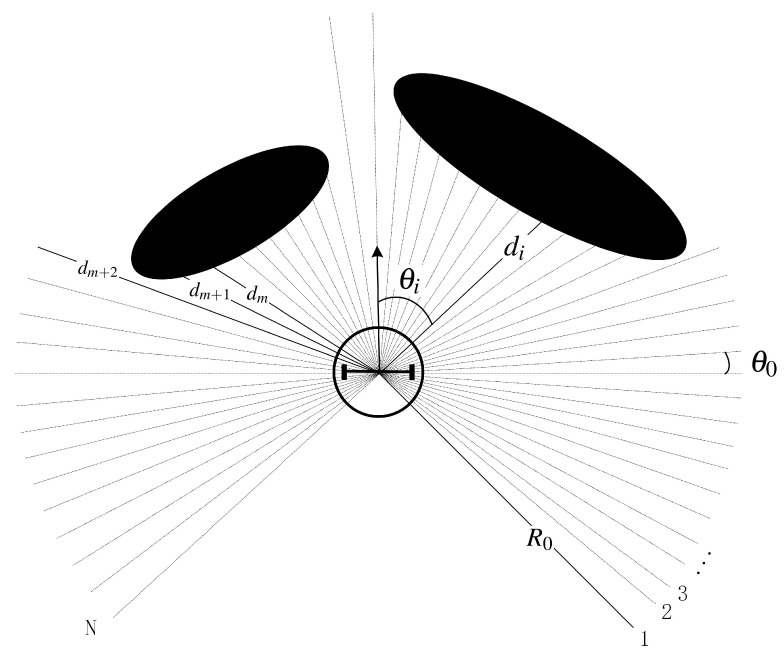
Representation of the LIDAR beams. The actual number of beams is much greater than illustrated here.

The sensor employed in the proposed approach is 2D LIDAR. The LIDAR is located at the center of the robot with a field of view (FOV) of $\theta_{FOV}$ (Figure 2). The LiDAR sends beams evenly within its entire FOV. The angle between two adjacent beams is referred as LIDAR resolution, denoted in this paper as $\theta_{0}$. $R_{0}$ is the farthest detection distance of the LIDAR. For each beam with the beam number *i*, $\theta_{i}$ and $d_{i}$ are the measured angle and the distance respectively. $d_{m},d_{m+1},d_{m+2}$ in Figure 2 is the measured distance of beam number m,m+1,m+2.

Define the heading of the robot as 0 degrees, then $\theta_{i}$ is calculated as:

(1) $$\begin{array}{c} \theta_{i}=i\theta_{0}-\theta_{FOV}/2 \end{array}$$

where i∈[1,N], with *N* being the total number of the LiDAR beams calculated as:

(2) $$\begin{array}{c} N=\theta_{FOV}/\theta_{0}+1 \end{array}$$

Similar methods that navigate a robot by identifying gaps using sensor data have been proposed in [17,18]. However, these methods will be invalid when faced with dense obstacles. In this paper, a more general method for identifying gaps is designed, which can find the appropriate gaps between densely interlaced obstacles.

Figure 2 shows that the value of $d_{m}$ and $d_{m+1}$ is very close, but there is a big difference between $d_{m+1}$ and $d_{m+2}$. Because the adjacent beams *m* and m+1 are directed at the same object, the smaller the LIDAR resolution is, the closer the values of $d_{m}$ and $d_{m+1}$ will be. Therefore, set a constant value $d_{T}$. When the difference between $d_{m}$ and $d_{m+1}$ is greater than $d_{T}$, we think that the adjacent beams *m* and m+1 are directed at two different objects and call the points where the two beams hit the objects as discontinuous points. At this time, the gap between these two objects may allow the robot to pass. Therefore, the first step is to find all the discontinuous points.

(1) Finding out the discontinuous points

**Figure 3:**
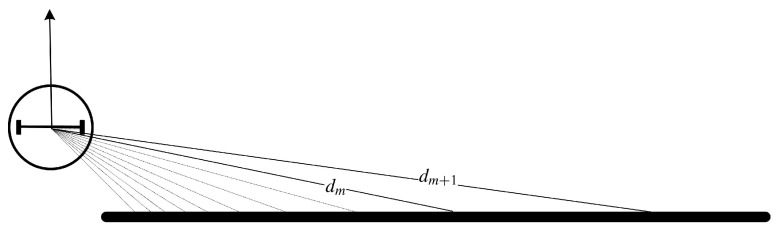
Representation of the LIDAR beams when the robot is near to a wall.

As mentioned above, there are discontinuous points when:

(3) $$\begin{array}{c} |d_{m}-d_{m+1}|>d_{T} \end{array}$$

where $d_{T}$ is constant. Obviously, $d_{T}$ should be larger than the diameter of the robot if the robot wants to pass between two discontinuous points. In theory, the value of $d_{T}$ should be as close to the robot diameter as possible because a larger $d_{T}$ may cause the loss of some discontinuities that allow the robot to pass. However, the LIDAR data $d_{m},d_{m+1}$ has deviations. If $d_{T}$ is equal to or close to the robot diameter, even though the discontinuous points satisfies $|d_{m}-d_{m+1}|>d_{T}$, the real distance between the two discontinuous points may be less than the robot diameter. On the other hand, the LIDAR beams are not continuous. The farther the obstacle is from the robot, the larger the difference between $d_{m},d_{m+1}$ is. When the robot is near a wall, the phenomenon will be more clear. This can be explained by Figure 3.

To show more clearly, the number of LIDAR beams in Figure 3 is much smaller than the actual number of beams. We can see that the difference between $d_{m}$ and $d_{m+1}$ is greater than the robot diameter. However, the adjacent beams *m* and m+1 are directed at the same object. So there are no openings to allow the robot to pass. At this time, setting $d_{T}$ to a larger value can avoid mistakenly identifying the discontinuous points. Therefore, when we set the value of $d_{T}$, there should be a trade-off between avoiding misidentification of discontinuities and avoiding loss of discontinuities. Also, More dense LIDAR beams can avoid misidentification of discontinuities. In the following experiments, the value of $d_{T}$ is obtained considering the error, resolution and farthest detection distance of the LIDAR.

(2) Making sure the discontinuous position is passable

**Figure 4:**
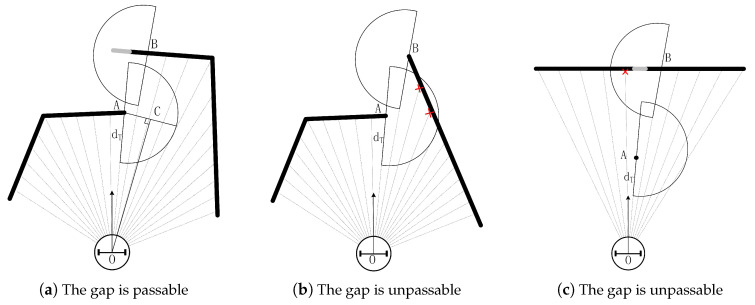
Examples of gaps. The black objects are obstacles that can be detected by LIDAR, and the gray objects are obstacles that cannot be detected.

Taking into account the size of the robot, the robot can pass only if the free space at the discontinuity is large enough. As shown in Figure 4, points A and B are discontinuous points that we found in the first step. There is a semicircle centered on point A on the right side of the line OA. If there are points detected by the LIDAR beams within the semicircle(shown in Figure 4b), we think that the robot cannot pass through the gap between point A and point B. As you can see in the picture, there is another semicircle centered at point B. The function of this semicircle is to eliminate the effects of LIDAR error data and extremely small obstacles. In Figure 4c, if there is a very small obstacle or error LIDAR signal at point A, checking whether there are points detected by the LIDAR beams within the semicircle at B can eliminate the interference. One LIDAR point is found in the semicircle centered on B, so there is no valid gap there.

**Figure 5:**
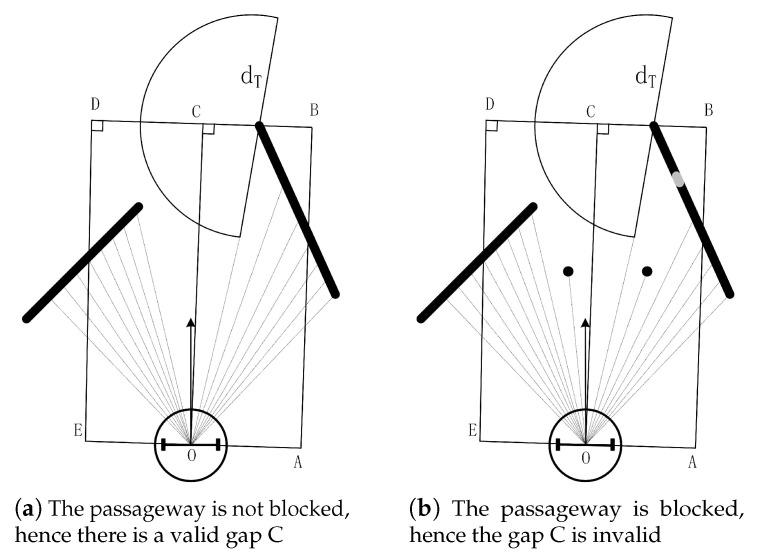
Examples of gaps. Rectangle CDEO and BCOA have a common side CD, and the lengths of sides CD and CB are equal to $d_{T}$.

There are no points detected by LIDAR beams within the two semicircles in Figure 4a. Therefore, the robot can pass through the gap between point A and point B. Point C is used to represent this gap, whose coordinates can be obtained by the fact that OC is the perpendicular bisector of the semicircle’s radius. Besides, the value of $d_{t}$ is set to be slightly larger than the diameter of the robot for safety considerations. If point A or point B exceeds the maximum range of LIDAR detection, then the semicircle corresponding to this point does not need to be considered, as shown in Figure 5.

(3) Making sure the passageway to the gap is not blocked

In the previous step, the gap that allows the robot to pass is found. However, moving directly toward the gap may be impracticable. As shown in Figure 5a, we find the gap by step (2). Nevertheless, after adding two point-like obstacles (Figure 5b), point C cannot be reached directly. Therefore, we cannot regard gap C in Figure 5b as a valid gap. To rule out this situation, we have defined two sets of points *M* and *N*. Point set *M* contains all the points detected by the LIDAR in the rectangle CDEO, and point set N contains all the points detected by the LIDAR in the rectangle BCOA. By checking gaps that found in the step (2) with the following conditions, we get the valid gaps that are not blocked:

(4) $$\begin{array}{cc} \; & d_{p_{1}p_{2}}>d_{T}\;\forall p_{1}\in M,\forall p_{2}\in N \end{array}$$

where $d_{p_{1}p_{2}}$ is the distance between $p_{1}$ and $p_{2}$. The method to obtain the elements of set M and N is to find the points that meet the following two conditions among the points detected by the LIDAR: (1) The distance from this point to the straight line OC is less than $d_{T}$. (2) The distance from this point to the robot center *O* is less than the length of the OC. The set determined by this method is slightly different from the set M and N defined before, but this dose not affect the research of this article.

By the above three steps, we can find all the valid gaps. The next part will show how to choose one of these gaps as a sub-goal.

### 3.2. Selecting Sub-Goal from Gaps

**Figure 6:**
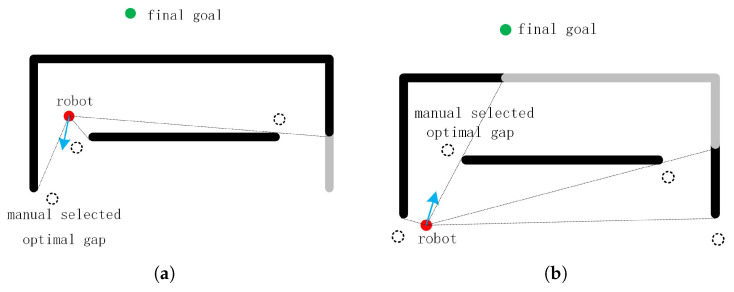
The identified gaps and manual selected direction of motion in two different positions.

Local navigation approaches are challenged by complex environments. For example, most of the local approaches cannot detour a big U-shaped obstacle. Some solutions [19,20] develop navigation strategies based on an empirical evaluation of trap situations. But they may still get stuck in some special environments. In [17], the author also adopted the concept of the gap, but the sub-goal was selected from gaps by the cost function method, which sometimes produced strange trajectories. Other local approaches, such as [21,22,23], did not solve the problem of navigation in complex environment. These local approaches have the common feature that they have no memory of the sensor date detected in the past. Our findings show that if one local planner does not take the past sensor data into account and there is no global planner available, then any improvement to the local planner cannot avoid being trapped by obstacles while considering the optimal path. Therefore, many existing local approaches cannot completely solve the trapped problem, even if they claim that they can avoid local minimum problems. This can be illustrated in Figure 6.

In Figure 6, the red point and green point are the robot position and the target position, respectively. Circles drawn with dotted lines represent the identified gaps. Firstly, we think that the two images are independent. Assuming that the robot can select the gap at the level of human intelligence, the optimal gap and motion direction is shown in Figure 6. However, the problem arises when Figure 6a is followed by Figure 6b. In Figure 6b, the robot arrives at the gap that selected in Figure 6a. Nevertheless, the manual selected optimal gap is close to the start point in Figure 6a, causing the robot to repeat the movement. Even if we derive the new sub-goal combining the current motion direction, the problem still exists.

To eliminate this defect, we have improved the traditional local approaches. When selecting a new sub-goal, the proposed approach combines the current sensor data with the previous sub-goals, which can effectively avoid the robot being trapped. The method we adopt is very simple, that is, the new sub-goal avoids the gaps that passed previously. In the remainder of this section, we will describe the method of selecting the sub-goal form these gaps.

In the previous part, we already know how to find out valid gaps. As shown in Figure 4a, there is a gap (indicated by point C). We find this gap since point A, point B, and its nearby LIDAR points satisfy those three conditions. The point that is closer to the robot between A and B is referred to as the origin of gap C. In order to avoid the robot being trapped because of continuous circular motion, the method we adopt is creating a blacklist to store the global coordinates of the origin of previously visited sub-goals (gaps). When selecting a new sub-goal, gaps whose origin is close to the point in the blacklist will not be taken into account. Furthermore, the origin of the sub-goal will be stored in the list only when the robot reaches this sub-goal and then leaves it a certain distance of $d_{list}$. In this way, the robot will not return to the gaps that have been to before.

**Figure 7:**
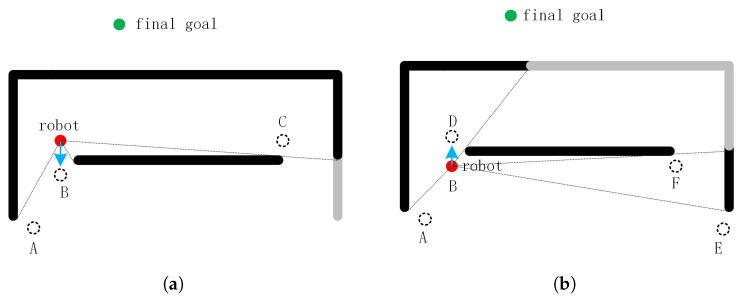
The direction of motion selected by a formula in two different positions.

In Figure 7a, the optimal sub-goal for manual selection is gap A. However, our robot does not have such a high level of intelligence. If a formula is used to calculate the optimal sub-target, the new problem comes out. For example, we can select the gap with the smallest value of $d_{sum}$ as the sub-goal:

(5) $$\begin{array}{c} d_{sum}=d_{r}+d_{g} \end{array}$$

where $d_{r}$ is the distance from the gap to the robot, $d_{g}$ is the distance from the gap to the goal. Obviously, the optimal sub-goal is gap B, see Figure 7a. Since the distance from the robot to the gap B or D is less than $d_{list}$, the origin of gaps B, D is not stored in the blacklist. Therefore the gap D cannot be ignored. However, gap D is selected as a new sub-goal since it has the smallest value of $d_{sum}$, see Figure 7b. The new sub-goal D is close to the starting point, causing the robot to repeat the movement.

To solve this problem, we add a new restriction to the sub-goal: the distance between the new sub-goal and the last sub-goal should be greater than the distance between the current position and the last sub-goal. For example, in Figure 7a, the starting point is treated as the first sub-goal, and the new sub-goal is gap B. In Figure 7b, the robot reaches gap B. The distance between gap D and the first sub-goal(the last sub-goal) is smaller than the distance between the current position B and the first sub-goal. Therefore, gap D can not be selected as the second sub-goal. In the remaining gaps, gap A is selected as the second sub-goal because of the minimum value of $d_{sum}$.

In short, the method of selecting sub-goal from gaps is divided into three steps.

(1) Exclude gaps whose origins are close to the point in the blacklist.
(2) Exclude gaps that are closer to the last sub-goal than the current position.
(3) Select the gap with the smallest value $d_{sum}$ as the new sub-goal.

### 3.3. Motion Control

Looking back at the flow chart in Figure 1, we can see that the sub-goal is not recalculated in each time interval. Only if the current sub-goal has been reached or is blocked, the sub-goal is updated. Methods of finding gaps and selecting sub-goal from gaps have been described in detail earlier. The location of the robot can be obtained by the equipped encoder. The cumulative error has little effect on the proposed method because the previous sub-targets that are farther away from the current position usually do not need to be considered and the previous sub-targets closer to the current position have small coordinates cumulative error. Therefore the global coordinates of the sub-goal can be derived, and it is easy to judge whether the sub-goal has been reached. The method of determining whether a sub-goal is blocked is described in Equation (4). Therefore, the last problem in Figure 1 is how to derive robot control commands based on the current sub-goal and LIDAR data.

In this paper, it is considered the wheeled mobile robot of unicycle type, in which the state variables are *x*, *y* (the world coordinates of the robot’s middle point), and θ (angle of the vehicle with the world X-axis). The kinematics of the robot can be modeled by:

(6) $$\begin{array}{c} \left\{\begin{array}{c} \dot{x}=\nu \cos\theta \;\;\;\\ \dot{y}=\nu \sin\theta \;\;\;\\ \dot{\theta }=\omega \;\;\;\;\;\; \end{array}\right. \end{array}$$

First, we design a piecewise control input. Once the current sub-goal is updated, a turning angle is then produced to drive the robot to face the direction of the sub-goal. At this time, the linear velocity ν is set to zero to avoid collisions:

(7) $$\begin{array}{c} \left\{\begin{array}{c} \nu =0\;\;\;\;\;\\ \omega =k_{2}atan\left(k_{3}\theta_{g}\right) \end{array}\right. \end{array}$$

When $\theta_{g}$ is close to zero, we can set the linear velocity ν to a fixed value and keep the robot moving towards the sub-goal:

(8) $$\begin{array}{c} \left\{\begin{array}{c} \nu =k_{1}\;\;\;\;\\ \omega =k_{2}atan\left(k_{3}\theta_{g}\right) \end{array}\right. \end{array}$$

where $k_{1},k_{2},k_{3}$ are three positive gains, $\theta_{g}$ is the angle of the sub-goal in the robot coordinate system.

In Equation (7), the robot rotates and does not collide. However, Equation (8) may cause collisions because of small obstacles and previously undetected obstacles. Therefore Equation (8) should be adjusted according to real-time LIDAR data.

**Figure 8:**
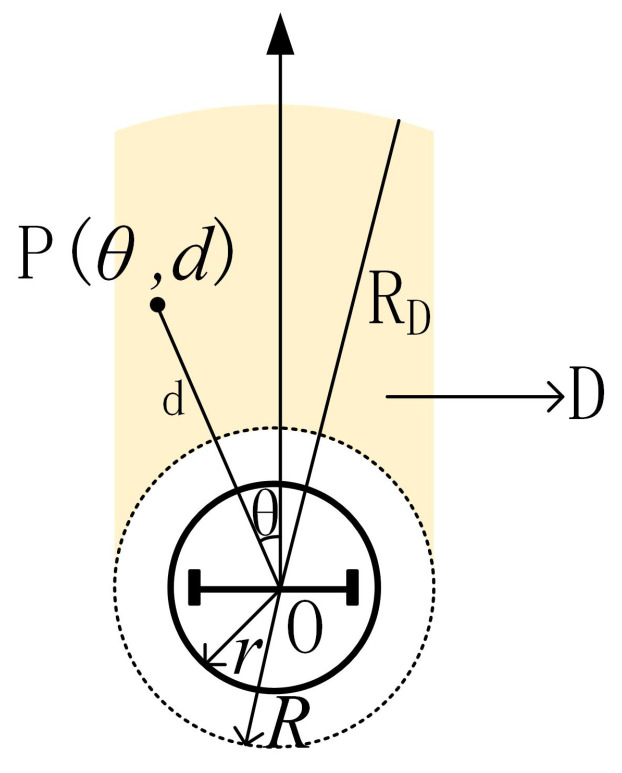
Representation of region *D*.

First of all, we define a region *D* (Figure 8) in the robot coordinate system. When obstacles are detected in area *D*, the control input (Equation (8)) should be changed accordingly.

Where *r* is the actual radius of the robot, *R* is the safe radius, which is larger than *r* to maintain a safe distance from the obstacle. The yellow area is *D*. The method of determining whether point P(θ,d) belongs to area D is given by:

(9) $$\begin{array}{c} P\left(\theta ,d\right)\in D\;\mathrm{if}\;R<d<R_{D}\;\mathrm{and}\;\left|d\sin\left(\theta \right)\right|<R\;\mathrm{and}\;\theta \in \left(-\frac{\pi }{2},\frac{\pi }{2}\right) \end{array}$$

**Figure 9:**
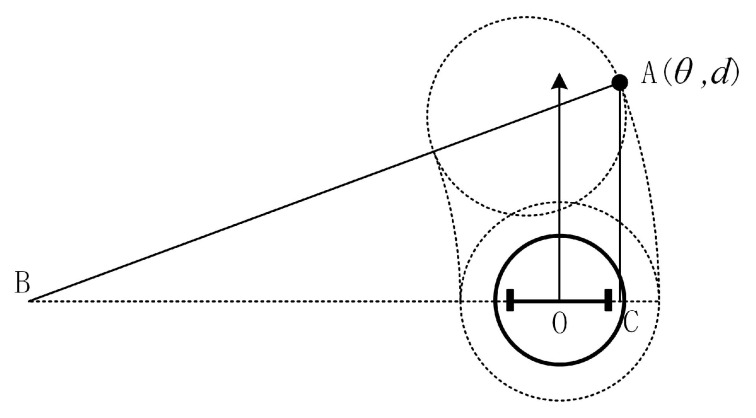
The robot passes by the left side of point-like obstacle A.

Then, consider a point-like obstacle, as shown in Figure 9.

Where point A is the detected obstacle and has polar coordinates (θ,d). If the robot passes from the left side of the point-like obstacle A and the trajectory is approximated as an arc, we can derive the radius $r_{BO}$ (Equation (10)) of the arc where the robot center is located according to the right triangle ABC. Since the robot’s linear velocity is fixed value ν, the value of angular velocity ω (Equation (11)) is derived in situation Figure 9.

$$\begin{array}{cc} \; & {\left(r_{BO}+R\right)}^{2}={\left(r_{BO}-d\sin\theta \right)}^{2}+{\left(d\cos\theta \right)}^{2} \end{array}$$

(10) $$\begin{array}{cc} ⟹ & r_{BO}=\left(d^{2}-R^{2}\right)/\left(2\left(R+d\sin\theta \right)\right) \end{array}$$

(11) $$\begin{array}{cc} ⟹ & \omega =\nu /r_{BO}=2\nu \left(R+d\sin\theta \right)/\left(d^{2}-R^{2}\right) \end{array}$$

Similarly, when the robot passes from the right side of the obstacle A, the angular velocity ω is calculated by:

(12) $$\begin{array}{c} \omega =\nu /r_{BO}=2\nu \left(-R+d\sin\theta \right)/\left(d^{2}-R^{2}\right) \end{array}$$

**Figure 10:**
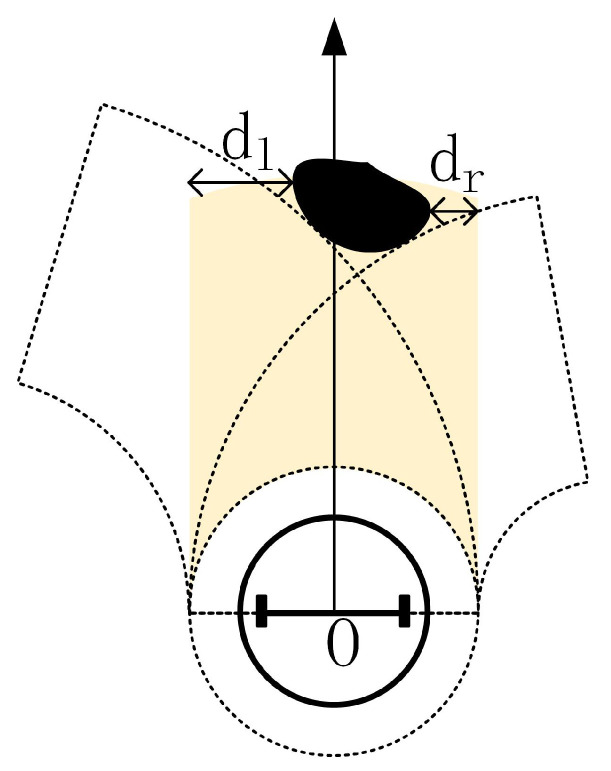
There is an obstacle in area *D*.

Generally speaking, real obstacles cannot be considered as a point. As shown in Figure 10, there is a black object in area *D* and multiple points (d,θ) are detected by LIDAR beams.

According to these detected points in area *D*, the distance from the obstacle to the left and right borders of area *D* can be calculated by:

(13) $$\begin{array}{c} d_{l}=\min\left\{R-d\cos\left(\theta \right)\;|\;\left(d,\theta \right)\in D\right\} \end{array}$$

(14) $$\begin{array}{c} d_{r}=\min\left\{R+d\cos\left(\theta \right)\;|\;\left(d,\theta \right)\in D\right\} \end{array}$$

For every point detected by LIDAR in area *D*, two angular velocities can be calculated using Equations (11) and (12). Therefore, the maximum value $\omega_{max}$ and the minimum value $\omega_{min}$ among these angular velocities can be derived. According to Equations (11) and (12), we can find that the value of ω can be very large when the distance *d* to the obstacle is close to the value of *R*. However, the angular velocities ω of the robot is bounded, and its maximum and minimum values are represented by $\omega_{1},\omega_{2}$, respectively. At this moment, the linear speed ν of the robot should be reduced to avoid the angular velocity ω exceeding the boundary. In short, in order to detour obstacles as shown in Figure 10, the following changes are made in contrast to Equation (8):

(15) $$\begin{array}{c} \left\{\begin{array}{lll} \omega =\omega_{max},\nu =k_{1} & \mathrm{if} & d_{l}\geq d_{r}\;\mathrm{and}\;\omega_{max}<\omega_{1}\\ \omega =\omega_{1},\nu =k_{1}\omega_{1}/\omega_{max} & \mathrm{if} & d_{l}\geq d_{r}\;\mathrm{and}\;\omega_{max}\geq \omega_{1}\\ \omega =\omega_{min},\nu =k_{1} & \mathrm{if} & d_{l}<d_{r}\;\mathrm{and}\;\omega_{min}\geq \omega_{2}\\ \omega =\omega_{2},\nu =k_{1}\omega_{2}/\omega_{min} & \mathrm{if} & d_{l}<d_{r}\;\mathrm{and}\;\omega_{min}<\omega_{2} \end{array}\right. \end{array}$$

$\omega_{max},\omega_{min}$ correspond to angular velocities of the robot just passing through the left side and right side of the obstacle. Is it possible that area D in the sub-target direction is entirely blocked by the obstacle causing $\omega_{max},\omega_{min}$ close to 0? No, because in each time interval, we will investigate whether the present sub-goal is blocked. If the current sub-goal is blocked, a new sub-goal will be selected.

To sum up, the steps of motion control in each time interval are as follows:

(1) If there are no obstacles in area D, control input is obtained by Equation (7) and (8). Otherwise, go to step (2) and (3)
(2) Calculate values of $d_{l},d_{r},\omega_{max},\omega_{min}$ using the points detected in area *D*.
(3) Replace Equation (8) with Equation (15).

## 4. Simulations and Experiments

### 4.1. Robotic Platform

The simulation and experimental platform for this novel approach is a wheeled differential drive robot, called Turtlebot2. We added a Hokuyo UST-20LX laser range finder to the robot. The sensor has a field of view of $270^{\circ }$ and a maximum scanning range of 10 m. The angular resolution is $0.25^{\circ }$ and 1080 measurements can be obtained per cycle. A laptop with an AMD A4-6210 APU processor running Ubuntu 16.04 is an onboard computer. The robot operating system (ROS) [24] running on the laptop is the middleware. Before the real tests of the proposed approach, the simulation was performed using the Gazebo/Rviz.

**Figure 11:**
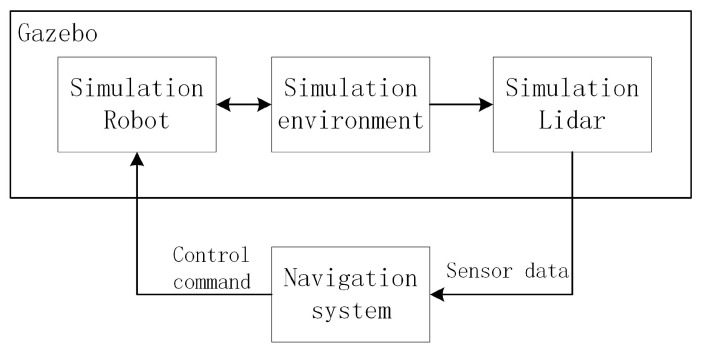
The simulation structure.

Gazebo is an open-source 3D robotic simulator, which integrated high-performance physics engines. It can provide simulation models for various scenarios in the real world. It can also model sensors, such as laser range finders and cameras. The simulation structure is shown in Figure 11. Gazebo models the robot, sensors, and environments. Rviz provides several plugins that display information such as images, models, paths, and more. The simulation software system is based on ROS, and each module communicates via ROS topic.

### 4.2. Further Implementation Details

The method proposed in this paper has been introduced in the second section. However, there are still many details in the experiment that have not yet been explained. The first question is the parameter settings. The parameters used during the experiment are given in Table 1.

Since it is not necessary to accurately reach the position of the sub-goal, when the distance between the present position and the sub-goal is less than 0.2, it is regarded as reaching the sub-goal. As shown in Figure 4a, this gap is represented by point C. When calculating the coordinates of point C, extend OC by 0.2 to compensate for the error. When selecting sub-goal from gaps, we established a blacklist. The gaps whose origin is close to the point in the blacklist will be excluded. In our experiments, that the distance to points in the blacklist is less than 0.6 is considered ’close’. In step (3) of selecting sub-goal from gaps, the gap with the smallest value $d_{sum}$ is selected as the new sub-goal. In fact, we found that choosing the gap with the smallest value of $d_{g}$ as a sub-goal can achieve better results. Also, as shown in Figure 1, in every control cycle, first considers if the final goal is blocked, if it is blocked, continue to move to the current sub-goal, otherwise go to the final target. The methods mentioned in this section are not unique. Maybe another solution is better, but the scheme we have adopted has already achieved our purpose well.

In this experiment, the equipped encoder is the only way to positioning. Even if there are cumulative errors, it has little effect on the approach. Because we only care about the coordinates of several recent gaps to prevent going back to the position we just passed. The position of the recent gaps has little cumulative error relative to the position of the robot.

### 4.3. Simulation Results

The avoidance of local minima is the main weakness of reactive based navigation approaches [25]. This leads to most existing navigation systems being dominated by global methods, while the reaction layer is simply a performer. The approach proposed in this paper stress on improving the local navigation to avoid being trapped by obstacles, so that the local algorithm can complete complex navigation tasks independently. Therefore, whether the robot can successfully reach the goal is the most concerning issue in this article.

**Figure 12:**
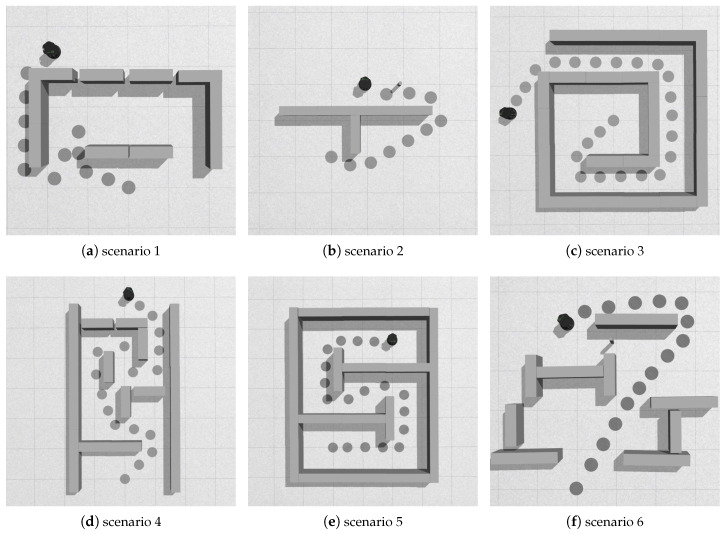
Examples of simulation.

Because the actual results of every obstacle avoidance algorithms are related to the parameter setting and other local approaches are difficult to reach the target in complex scenarios, hence in the simulation part, we did not compare the proposed approach with other methods. As complex environments can be more easily constructed in simulation environments, extensive simulation studies were carried to prove the effectiveness of the new method. Figure 12 shows six typical simulation scenarios, which are the screenshots of the simulation after the robot reaches the target. Since the gazebo software itself cannot display the motion path of the robot, we manually mark where the robot passed with grey circles. The position of the robot in the picture is the final target.

First, look at scenario 1, we build a scene similar to the one in Figure 6, and the robot arrives at the target successfully. Because our method takes into account the size of the robot, small openings between the obstacles are not identified as valid gaps. In scenarios 2 and 3, the robot can pass concave obstacles. In scenario 2, after finding the sub-goal point, the robot could adjust the motion control in time because of the cylindrical obstacle. Scenarios 4, 5, and 6 are more complicated. The approach proposed in this paper successfully reached the target in the six scenarios, and the resulting path is close to the optimal path. But these are challenging tasks for other local approaches.

### 4.4. Real Robot Experiments

**Figure 13:**
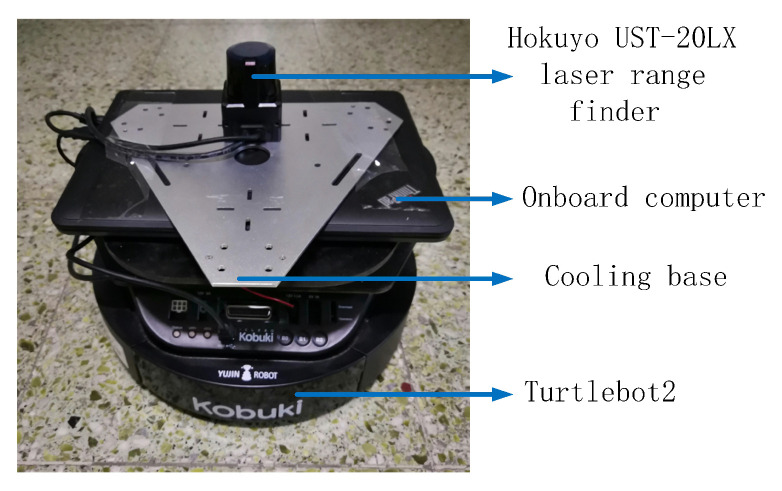
Turtlebot2.

To further verify the applicability of the new navigation algorithm proposed in this paper, we have implemented it on a real Turtlebot2 robot. Other hardware and parameters in the real experiment and the simulation test are the same. The advantage is that the simulation results are basically the same as the real experimental results. Because both simulation and experiment are based on ROS, the simulation code is easy to transplant to the real robot. Figure 13 shows the robot we use.

**Figure 14:**
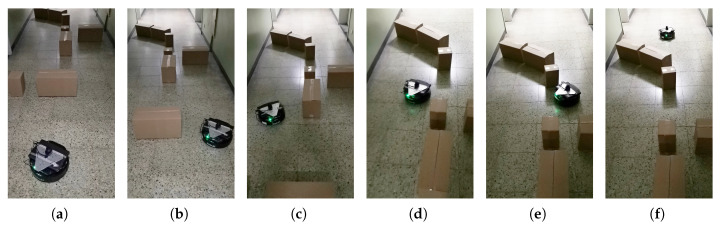
The scenario of real experiment.

**Figure 15:**
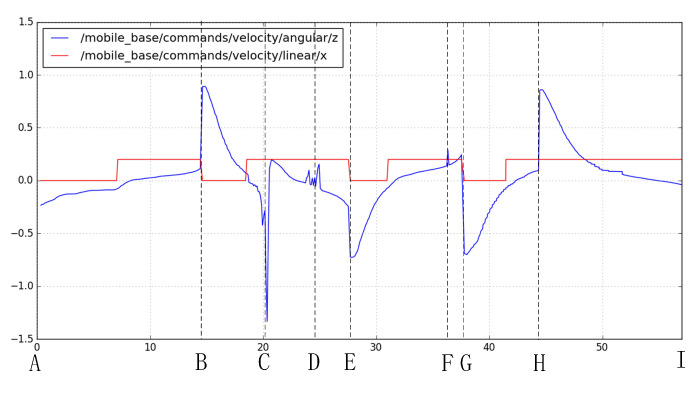
Linear velocity (**red**) and the angular velocity (**blue**) of the robot during the experiment.

The goal was set 6 m in front of the robot. Figure 14 illustrates the environment where the experiments were performed. Figure 15 shows linear velocity and the angular velocity of the robot during the experiment. The horizontal axis represents time. In addition we marked 9 positions (A–I) in Figure 15.

At time A, B, E, G in Figure 15, the robot’s reached the current sub-goal and found a new sub-goal. At this time, the robot’s linear velocity was zero and turned to the new sub-goal, corresponding to Figure 14a–d. At time H, the final target was found, and we did not set linear velocity to zero, corresponding to Figure 14e. At the time I, the robot reached the final target, corresponding to Figure 14f. At time C, D, F, angular velocity fluctuation is the result of Equation (15). LIDAR points in area D are detected because of LIDAR noise or obstacles in front. Thus angular velocity is adjusted.

## 5. Discussions

In order to verify our method, many experiments were carried out. In most of the simulation tests, satisfactory results have been derived. However, there are some cases of navigation failure. For example, when the robot and the wall are almost collinear, the LIDAR detected points on the wall are sparse, resulting in wrongly identified gaps. Sensor noise may also cause gaps to be identified incorrectly. On the other hand, there are also cases where some gaps are not recognized. In addition, when it comes to moving obstacles, if the optimal sub-goal changed due to the movement of obstacles, the proposed method has the problem of not being able to update the optimal sub-goal in time. These problems may cause the path to be longer or even the target to be unreachable. Though we cannot guarantee that if a solution exists our method will find it, the experiment achieved satisfactory results. The problems in this article will be further studied in the future.

## 6. Conclusions

This paper presented a sub-goal seeking approach for local navigation in complex, unknown environments, which can identify gaps and select one of the gaps as a sub-goal to guide the robot to move toward the target. This paper has two main contributions. One is to design a clever gap recognition method, and the other is finding the common defect of the local algorithms and making improvements. It can avoid the robot from being trapped in complex environments so that the local algorithm can complete complex navigation tasks independently and no longer depends on the global algorithms. Therefore, the proposed approach has low computational complexity, which is of great significance to robots with low computing power. And it will also help to reduce the cost of robots. Finally, simulations and real tests are performed. The results suggest that our approach is robust in controlling the robot and can reach the goal in various environments.

## Figures and Tables

**Table 1:** The parameters in the experiment.

Parameter	Significance	Value
*r*	The radius of Turtlebot2	0.17 m
$d_{T}$	The parameter in Equation (3)	0.6 m
$k_{1}$	The parameter in Equation (8)	0.2
$k_{2}$	The parameter in Equation (7)	1.5
$k_{3}$	The parameter in Equation (8)	1
*R*	The safe radius *R* of the robot in Figure 8	0.25 m
$R_{D}$	The radius of region D in Figure 8	0.5 m
